# PsychoPharm aggregated risk score (PARS): a multidimensional tool to flag high-risk pharmacotherapy in psychiatric patients

**DOI:** 10.3389/fphar.2026.1782968

**Published:** 2026-04-01

**Authors:** Florina-Diana Goldiş, Sabina-Oana Vasii, Sebastian-Mihai Ardelean, Mihai Udrescu-Milosav, Lucreția Udrescu

**Affiliations:** 1 Center for Drug Data Analysis, Cheminformatics, and the Internet of Medical Things, Victor Babeş University of Medicine and Pharmacy Timişoara, Timişoara, Romania; 2 Doctoral School of Pharmacy, Victor Babeş University of Medicine and Pharmacy Timişoara, Timişoara, Romania; 3 Department of Computer and Information Technology, Politehnica University Timişoara, Timişoara, Romania; 4 Department of Clinical Pharmacy and Drug Analysis, Victor Babeş University of Medicine and Pharmacy Timişoara, Timişoara, Romania

**Keywords:** anticholinergic cognitive burden, composite risk score (PARS), drug-drug interactions, logistic regression, polypharmacy, psychopharmacotherapy, QTprolongation, serotonergic agents

## Abstract

**Introduction:**

Psychiatric outpatients frequently receive complex, long-term regimens where polypharmacy, drug-drug interactions (DDIs), QT interval risk, anticholinergic load, and serotonergic exposure co-occur. Clinicians must triage drug lists quickly, yet most tools address a single risk domain. We developed the PsychoPharm Aggregated Risk Score (PARS), a visit-level composite score that integrates multiple pharmacological risk domains into a single, clinically interpretable signal.

**Methods:**

We analyzed 2,666 visits from 680 adults in ambulatory psychiatry. For each visit, we computed the risk components: DDI score from DrugBank, AZCERT QT risk, anticholinergic cognitive burden (ACB), serotonergic exposure, and polypharmacy ( ≥ 5 drugs). Using z -standardized components, we created the PARS composite as their equal-weight mean, and report it as PARS rescaled on a 0–10 scale for clinical communication. We trained a logistic regression to estimate the probability of High-risk PARS, defined as PARS ≥ 75th percentile, using only prescribed drugs, age, and sex as features (explicitly excluding the engineered components to prevent leakage), then compared with additional stratifications by age and sex, as well as a 5-fold GroupKFold. A fixed decision threshold of 0.30 was set to favor recall and F1 for the positive class. A drug-only model was also used to identify drugs associated with High-risk visits.

**Results:**

The prevalence of high-risk cases was observed in 25.2% of visits. Across validation schemes, the primary 80–20 patient-level split achieved the best accuracy, precision, and F1 at the 0.30 operating point. In the primary 80–20 split stratified by High-risk status, the logistic model achieved an AUC of 0.931 (patient-bootstrap 95% CI 0.875–0.971). Discrimination was similar with additional age and sex stratification (AUC of 0.938) and 5-fold GroupKFold (pooled out-of-fold AUC 0.939), indicating robustness to partitioning. In the drug-only model, positive associations included drugs such as quetiapine, haloperidol, clozapine, and amiodarone. The highest-ranked visits combined central nervous system-heavy regimens and polypharmacy.

**Conclusion:**

As an exploratory tool, PARS integrates DDIs, QT risk, anticholinergic cognitive burden, serotonergic exposure, and polypharmacy into a single probability that reliably discriminates High-risk visits and supports screening at a 0.30 operating threshold. Our approach highlights actionable drug combinations and patient profiles for drug review and deprescribing.

## 1 Introduction

Psychoactive drugs are among the most frequently prescribed medications, due to the rising prevalence of mental illness Marović et al. (2024). Psychiatric patients often receive complex, multi-drug regimens for extended periods, making polypharmacy common and clinically consequential Wolff et al. (2021). There is an increase in the number of prescribed drugs, as psychiatric patients have clinical situations that require this approach; they may have diseases associated with mental disorders and, thus, will require additional treatments for these conditions Wolff et al. (2021); Dubrall et al. (2025). Few psychiatrists are aware of the comorbid drug of the patients they treat Dornquast et al. (2017).

Polypharmacy definitions vary; a widely used operational definition is the concurrent use of ≥ 5 drugs Stassen et al. (2022); Masnoon et al. (2017). Polypharmacy can also refer to the administration of two medications over a period longer than 240 days Masnoon et al. (2017). Major polypharmacy involves the use of ten or more drugs, while hyperpolypharmacy means taking ten or more drugs for at least 90 days. Persistent polypharmacy is characterized by the use of at least five drugs for a minimum of 181 days. Chronic polypharmacy refers to the use of ≥ 5 medications for 1–6 months within a year.

Psychiatry adds specific patterns such as same-class and multi-class polypharmacy, while total polypharmacy further expands to all drugs as co-treatment of somatic comorbidities Carmona-Huerta et al. (2019). Antipsychotic polypharmacy defines prescribing two or more antipsychotics for more than 14 days Ganguly et al. (2004); Foster and King (2020).

Polypharmacy increases the risk of drug-drug interactions (DDIs), which can alter plasma concentrations or therapeutic activity and lead to toxicity or reduced efficacy Dubrall et al. (2025); Marović et al. (2024). In psychiatric practice, DDI risk is amplified by chronic courses, relapses, and combinations of psychotropics with somatic therapies Preskorn (2018); Al Zaabi et al. (2020); pharmacovigilance data suggest a high proportion of major and even contraindicated DDIs among adult psychiatric patients Dubrall et al. (2025).

Mechanistically, interactions arise through pharmacokinetics (notably CYP450 isoenzymes such as CYP3A4, 2D6, 2C9, 1A2, 2C19, and 2E1) Moreau et al. (2021); Hefner et al. (2020); Daniel et al. (2022); Franz et al. (2012); de Leon et al. (1998) and pharmacodynamics (additive/synergistic or antagonistic receptor effects) Aiken (2021); Reeve et al. (2025). Many psychotropics are CYP inducers/inhibitors and share metabolic pathways with widely used somatic drugs (e.g., β -blockers), while patient factors (age, liver and renal disease, genetic polymorphisms) further modulate risk Franz et al. (2012); Moreau et al. (2021); Daniel et al. (2022).

Two pharmacodynamic domains are particularly salient in psychiatry: QT interval prolongation (and torsades de pointes risk) and anticholinergic cognitive burden. AZCERT (CredibleMeds) classifies drugs by known, possible, or conditional risk of torsades Woosley et al. (2025); co-prescriptions of QT-prolonging agents are common in psychiatric cohorts. Literature reports that a substantial fraction of psychiatric patients are exposed to QT-prolonging DDIs Ramasubbu et al. (2024); Meid et al. (2017); Hefner et al. (2021); Das et al. (2019), with a non-negligible proportion showing ECG changes Das et al. (2021). Risk is heightened in older adults and by combinations such as antipsychotic with antidepressant or multiple QT risk drugs Ray et al. (2009). Anticholinergic cognitive burden (ACB), prevalent due to psychotropics with antimuscarinic properties, is associated with cognitive impairment, functional decline, and worse long-term outcomes in psychotic disorders Al Rihani et al. (2021); Peralta et al. (2024); Penttilä et al. (2005).

In addition, serotonin syndrome—a clinical diagnosis resulting from serotonergic toxicity—can occur with selective serotonin reuptake inhibitors (SSRIs), serotonin-norepinephrine reuptake inhibitors (SNRIs), tricyclic antidepressants (TCAs), certain anticonvulsants, analgesics (e.g., tramadol, fentanyl), antimicrobials (e.g., linezolid), and even herbal products (e.g., St. John’s wort). Incidence may rise with increasing antidepressant use, and moderate cases are often missed, underscoring the need for proactive detection Foong et al. (2018); Mikkelsen et al. (2023).

Collectively, these factors—ageing, comorbidity, DDI-prone pharmacotherapy, QT risk, anticholinergic load, and serotonergic exposure—create a real-world need for integrative, clinically usable risk tools to support medication review, deprescribing, and monitoring decisions in psychiatric care.

In this context, we aimed to develop and validate the PsychoPharm Aggregated Risk Score (PARS), a composite, visit-level score that integrates (i) DDI severity (according to DrugBank), (ii) AZCERT QT risk categories, (iii) ACB anticholinergic cognitive burden, (iv) exposure to serotonergic drugs, and (v) polypharmacy status, to estimate the probability that a visit is High-risk and to assist clinical screening and medication optimization. To this end, our main objectives are:

- To build component metrics per visit based on DDIs, AZCERT, ACB, serotonergic exposure, and polypharmacy, and aggregate them into PARS and a 0–10 PARS rescaled for clinical communication.
- To train a logistic model to estimate High-risk PARS probability using only prescribed drugs, age, and sex as features (explicitly excluding engineered components to prevent leakage); evaluate performance with patient-level 80–20 splits (stratified by having ≥ 1 High-risk visit, and additionally by age and sex) and GroupKFold 5-fold validation.
- To identify drugs most strongly associated with High-risk PARS and profile patient-level patterns (e.g., recurrent high PARS vs. isolated spikes) that may inform deprescribing, ECG and electrolyte monitoring, and risk communication.

Together, these considerations motivate PARS as a pragmatic, data-integrated screening tool; in what follows, we detail its construction and validation, and evaluate its clinical operating point for routine use.

## 2 Materials and methods

### 2.1 Study design

We conducted a retrospective observational study on a dataset of outpatients attending a private ambulatory psychiatry clinic in Timişoara, western Romania, during January–December 2023. The clinic provides longitudinal outpatient mental healthcare, with patients typically followed at regular intervals (often monthly) for clinical reassessment, treatment adjustments, and prescription renewal; accordingly, multiple visits per patient are recorded. We included only the following categories of patients: (1) those with a minimum of two documented medications, (2) individuals aged 18 years or older, and (3) those who provided signed informed consent. Since the patients included in our study had one or more documented visits, we used the visit data as the unit of analysis. All patient data was de-identified before analysis. The study protocol was reviewed and approved by the Scientific Research Ethics Committee of the “Victor Babeş” University of Medicine and Pharmacy Timişoara (approval no. 59/16.12.2022 rev). The patients provided their written informed consent to participate in this study; this was obtained during clinic visits in 2023, before data extraction and analysis.

### 2.2 Data and variables

The data recorded at the visit level for each patient included: visit ID, patient ID, date, sex, age, diagnosis, and the list of drugs per visit.

For each visit, we assessed drug-drug interactions (DDIs) using DrugBank and counted pairs for each category of returned result (number of major DDIs, number of moderate DDIs, number of minor DDIs, and number of pairs for which the result was No Interactions Found) Knox et al. (2024).

We flagged each visit based on the polypharmacy profile (i.e., 5 or more drugs) Stassen et al. (2022); Masnoon et al. (2017).

We also used drug lists, including AZCERT for the QT prolongation risk Woosley et al. (2025), ACB for anticholinergic cognitive burden King and Rabino (2024), and those associated with serotonin syndrome risks Knox et al. (2024).

### 2.3 Visit-level risk components

#### 2.3.1 Drug-drug interaction severity

For each visit, we calculated an aggregated DDI severity score based on DrugBank, using Equation 1:

$${DDIscore}_{i}=3\cdot N_{i}^{\text{maj}}+2\cdot N_{i}^{\text{mod}}+1\cdot N_{i}^{\text{min}},$$ (1)

where $N_{i}^{\text{maj}}$ , $N_{i}^{\text{mod}}$ , and $N_{i}^{\text{min}}$ represent the number of drug pairs of major, moderate, and minor DDIs, respectively.

#### 2.3.2 Polypharmacy

We evaluated each visit for the presence of polypharmacy (see Equation 2), scoring it with a 1 if the set of unique drugs $D_{i}$ included five or more drugs, or with a 0 if not:

$$\text{Has polypharmacy}=\left\{\begin{array}{c} 1,\;\text{if }D_{i}\geq 5\\ 0,\text{otherwise}. \end{array}\right.$$ (2)

#### 2.3.3 QT risk

We used the AZCERT list of drugs classified according to their potential to cause QT prolongation or torsades de pointes (TdP). We assigned each drug a score according to the corresponding risk class, as outlined in Equation 3:

$$s\left(d\right)=\left\{\begin{array}{cc} 3, & \text{if AZCERT category is Known}\\ 2, & \text{if AZCERT category is Possible}\\ 1, & \text{if AZCERT category is Conditional}\\ 0, & \text{otherwise.} \end{array}\right.$$ (3)

We derived two scores: ${AZCERTmax}_{i}$ , which is the highest AZCERT score among all the patient’s drugs per visit (Equation 4), and ${AZCERTsum}_{i}$ , which is the sum of all AZCERT scores for each visit (Equation 5):

$${AZCERTmax}_{i}=\underset{d\in D_{i}}{\max}s\left(d\right),$$ (4)

$${AZCERTsum}_{i}=\underset{d\in D_{i}}{\sum }s\left(d\right),$$ (5)

where $D_{i}$ denotes the set of unique drugs recorded at visit i and s(d)∈{0,1,2,3} is the per-drug AZCERT mapping.

Our predictive model relies on ${AZCERTmax}_{i}$ as the primary QT signal, while ${AZCERTsum}_{i}$ is provided for descriptive purposes.

#### 2.3.4 anticholinergic cognitive burden

The anticholinergic cognitive burden (ACB) assesses the risk of cognitive impairment associated with certain medications. Drugs that are known to have a definite anticholinergic cognitive burden are assigned a score of 2 or 3, while those with a possible anticholinergic cognitive burden receive a score of 1. Drugs that are not found in the ACB lists are treated as 0. Using Equation 6, we calculated the ACB score for each visit by summing the ACB scores of the drugs in the set $D_{i}$ of unique drugs.

$${\text{ACB score visit}}_{i}=\underset{d\in D_{i}}{\sum }ACB\left(d\right).$$ (6)

#### 2.3.5 Serotonin syndrome risk

We downloaded the list of approved serotonergic drugs from DrugBank. Equation 7 defines SERO count, the number of serotonergic agents recorded at visit i . Equation 8 derives the visit-level flag SERO any, which indicates whether at least one serotonergic drug was present (1 = yes, 0 = no). If a drug is not listed under serotonergic substances, it is considered as 0.

$${\text{SERO count}}_{i}=\underset{d\in D_{i}}{\sum }SERO\left(d\right),$$ (7)

$${\text{SERO any}}_{i}={\text{SERO count}}_{i}\geq 1.$$ (8)

### 2.4 Building the PARS aggregate score

For each visit i we used 5 components: DDIscore, Has polypharmacy, AZCERT max, ACB score visit, and SERO any. For each component X , we calculated the mean $\mu_{X}$ and standard deviation $\sigma_{X}$ across all visits and standardized to z -scores using Equation 9,

$$z_{i}\left(X\right)=\frac{X_{i}-\mu_{X}}{\sigma_{X}},$$ (9)

where X∈{DDIscore,Has polypharmacy,AZCERTmax,ACB score visit,SERO any}.

The PsychoPharm Aggregated Risk Score (PARS) for visit i is the average of these five z -scores (Equation 10):

$${\text{PARS}}_{i}=\frac{z_{{\text{DDIscore}}_{i}}+z_{{\text{Has polypharmacy}}_{i}}+z_{{\text{AZCERTmax}}_{i}}+z_{{\text{ACB score visit}}_{i}}+z_{{\text{SEROany}}_{i}}}{5}.$$ (10)

The meaning of the PARS score is as follows:

- PARS = 0, risk around the cohort mean,
- PARS > 0, risk above the cohort mean,
- PARS < 0, pharmacological risk under average.

Equal weights were used as a baseline because no validated differential weights are available for these domains in psychiatric polypharmacy without endpoint anchoring Searle et al. (2008); Barclay et al. (2019); Greco et al. (2019).

For clinical display, we rescaled PARS to a 0–10 range using min -max scaling, as shown in Equation 11.

$${PARSrescaled}_{i}=10\cdot \frac{{PARS}_{i}-{PARS}_{\min}}{{PARS}_{\max}-{PARS}_{\min}}.$$ (11)

Here, ${PARS}_{\min}=\min_{j}{PARS}_{j}$ and ${PARS}_{\max}=\max_{j}{PARS}_{j}$ are computed over the reference set (e.g., the training set) to avoid leakage.

A PARS rescaled of 0 denotes the lowest observed PARS in the reference cohort, while 10 corresponds to the highest observed PARS. This transformation is monotonic; it does not change visit ranking.

To assess potential redundancy between polypharmacy ( ≥ 5 drugs) and DDI burden, we examined correlations among PARS components and quantified variance inflation factors (VIFs). We additionally evaluated the frequency of discordant visits (polypharmacy with low DDIscore, and non-polypharmacy with high DDIscore) as an empirical check of construct overlap.

### 2.5 Defining the high-risk PARS binary label

To provide a screening label proportional to relative risk in the cohort, we defined a binary visit-level outcome using the empirical distribution of PARS. Let $\tau_{75}$ denote the 75th percentile of PARS computed on the reference set (e.g., the training set during model development). We set in Equation 12:

$${\text{High}-\text{risk PARS}}_{i}=\left\{\begin{array}{c} 1,\;\text{if }{PARS}_{i}\geq \tau_{75},\\ 0,\text{otherwise}. \end{array}\right.$$ (12)

This visit-level label serves as the ground truth for training and evaluating the predictive model. Based on this definition, the expected prevalence is approximately 25% (considering possible ties at $\tau_{75}$ ). By contrast, a probability cutoff (e.g., ${\hat{p}}_{i}\;\geq$ 0.30) is an operating threshold applied to the predicted probability $\hat{p}$ (High-risk = 1) to generate a binary screening alert.

### 2.6 Drug-based logistic model for predicting high-risk PARS

To identify drugs most strongly associated with High-risk PARS visits, we trained an L2-regularized binary logistic regression (scikit-learn, Python) that predicts the High-risk PARS label using only prescribed drugs, age, and sex.

#### 2.6.1 Feature set for prediction

For each visit, we built a sparse binary vector with one column per unique active substance (1 = present during the visit, 0 = absent), plus age and sex. The demographics added to the design matrix included age, recorded as a continuous variable, and sex, represented as female or male (F = 1, M = 0). To prevent information leakage, we excluded all engineered PARS components from the predictors (DDIscore, Has polypharmacy, AZCERTmax, ACB score visit, and SEROany), as well as PARS itself.

#### 2.6.2 Splitting and validation strategies

We applied two strategies to run the logistic regression:

- Train-test 80–20 (stratified by High-risk PARS). We split patients 80%/20% into train/test at the patient level. Stratification used a patient-level indicator of having ≥ 1 High-risk PARS visit (yes/no) to preserve outcome prevalence across splits.
- Train-test 80–20 with combined stratification (age + sex + High-risk PARS). Per patient, we formed a combined stratification label using age ( < 65 and ≥ 65), sex (female–F and male–M), and high-risk status (has and has not ≥ 1 High-risk PARS visit). This way, we obtained 8 strata used in stratify to balance demographics and risk across train/test.

The logistic model produces an estimated probability ${\hat{p}}_{i}$ for each visit i (Equation 13):

$${\hat{p}}_{i}=\mathrm{Pr}\;\left({\text{High}-\text{risk PARS}}_{i}=1\;|\;{\text{features}}_{i}\right).$$ (13)

To assess the robustness of the logistic model, we performed a 5-fold GroupKFold cross-validation. We used GroupKFold ( k = 5), using patient ID as the grouping variable, so that all visits from a given patient reside in the same fold; each fold served once as validation while the remaining four formed the training set.

To assess whether the operating threshold could be influenced by tuning, we compared the pre-specified threshold (0.30) with thresholds selected only on the training set using two standard criteria: (i) maximization of F1 and (ii) the Youden index (J=recall+specificity−1) , and evaluated all thresholds on the held-out test set.

Model performance was assessed on the held-out test set (patient-level 80–20 splits) and on each validation fold (5-fold GroupKFold). We computed ROC-AUC (threshold-independent discrimination), accuracy, precision, recall, and F1 score for the positive class (High-risk PARS=1) at the operating threshold (0.30). For interpretability, we report model coefficients and exponentiated odds ratios (OR) for drug indicators, alongside a cohort-level support measure (the number of visits in the full cohort in which each drug was present). The highest-ranked drugs by coefficient magnitude are derived from the model trained under the primary patient-level 80–20 split; to avoid information leakage, all visits from a given patient were confined to a single split. OR 95% confidence intervals were obtained using patient-level bootstrap resampling on the training set (2000 replicates).

We assessed probabilistic calibration using the Brier score and calibration plots (reliability diagrams) with quantile binning (10 bins). For GroupKFold, calibration was computed from pooled out-of-fold predictions to avoid optimistic bias (see Supplementary Material).

We employed Python 3.12.7 using Pandas and NumPy for data preprocessing and Scikit-Learn for modeling and validation (pipeline, logistic regression, and GroupKFold). All train-test splits were generated with fixed random seeds to ensure reproducibility. To prevent data leakage, transformers and scalers were fitted exclusively on the training data within each split and cross-validation fold, and then applied to the corresponding held-out data.

## 3 Results

### 3.1 Cohort description

Table 1 displays demographic characteristics, and medical service usage; the data are presented as counts, along with means, standard deviations, and interquartile ranges (minimum-maximum). The analysis included a total of 2,666 office visits, corresponding to 680 unique patients. Of these, 68.5% were women and 31.5% were men. The mean age of the patients was 60.1 ± 18.3 years, with a median of 62 years (IQR 46.4–74.0). In our cohort, women were slightly older (mean 61.8 years, IQR 50–75) compared to men (mean 57.5 years, IQR 44–71.8). The number of visits per patient ranged from 1 to 13, with a median of 2.5 visits/patient. At the age level, 54.5% were < 65 years, and 45.5% were ≥ 65 years old. 25.2% of visits were classified as High-risk PARS, according to the probability threshold of 0.30 (Section 3.2; Table 3). At the patient level, 31.0% had at least one visit classified as high pharmacological risk, indicating that approximately one-third of the included patients experienced, at least once, a therapeutic regimen considered high-risk according to the PARS score.

**TABLE 1:** Cohort characteristics.

Characteristic	Value	Level
Demographics		
Patients, total (N)	680	​
Sex, n (%)	Female 466 (68.5%)	Patient-level
	Male 214 (31.5%)	Patient-level
Age, mean ± SD (years)	60.1 ± 18.3	Patient-level
Age, median (IQR), years	62 (46.4–74.0)	Patient-level
Age range, years	18–97	Patient-level
Age group, n (%)	<65 : 372 (54.7%)	Patient-level
	≥65 : 308 (45.3%)	Patient-level
Service use		
Visits, total (N)	2,666	Visit-level
Median visits per patient (min–max)	2.5 (1–13)	Patient-level
Patients with exactly 1 visit, n (%)	238 (35.0%)	Patient-level
Patients with ≥2 visits, n (%)	442 (65.0%)	Patient-level
Exposure to high PARS-defined risk		
High-risk PARS visits, n (%)	671 (25.2%)	Visit-level
Patients with ≥1 high-risk PARS visit, n (%)	211 (31.0%)	Patient-level

The distribution of the pharmacological risk components that feed PARS is presented in Table 2. Across visits, the DDI severity score ranged between 0 and 136, with a mean of 15.62 ± 17.99. Anticholinergic cognitive burden per visit (ACB) was modest (mean 2.51 ± 2.01 and IQR 1–4). However, anticholinergic cognitive burden was frequent at clinically meaningful levels: 41.0% of visits encountered ACB score visit ≥ 3, and 14.9% achieved ACB ≥ 5, indicating that a substantial proportion of visits involved moderate-to-high anticholinergic exposure. The AZCERTmax per visit had a median of 2.0, which indicates that the typical visit has an AZCERTmax labeled as Conditional; the IQR range 1.0–2.0 reflects that 50% of visits fall between Possible and Conditional; the AZCERTmax score per visit was typically Possible/Conditional (mean 1.56 ± 0.80). The aggregate PARS ( z -score) centered near zero (mean 0.00 ± 0.64; median − 0.02), with its 0–10 rescaled version averaging 3.25 (median 3.19). Regarding binary indicators, 83.5% of visits contained at least one serotonergic drug, 48.6% met the polypharmacy criterion ( ≥ 5 drugs), and 25.2% were labeled High-risk PARS. These figures provide context for the operating characteristics reported later and illustrate that high-risk visits represent roughly one-quarter of the cohort.

**TABLE 2:** Descriptive statistics for pharmacological risk components and PsychoPharm Aggregated Risk Score (PARS) at the visit level, displayed with standard deviation (SD), and interquartile range (IQR).

Risk components/scores	Mean ± SD	Median (IQR)	Min–Max
DDIscore	15.62 ± 17.99	8.50 (3.00–21.00)	0.00–136.00
ACB score visit	2.51 ± 2.01	2.00 (1.00–4.00)	0.00–14.00
AZCERTmax	1.56 ± 0.80	2.00 (1.00–2.00)	0.00–3.00
PARS	0.00 ± 0.64	− 0.02 ( − 0.32–0.39)	− 1.46–3.03
PARS rescaled	3.25 ± 1.43	3.19 (2.53–4.12)	0.00–10.00

Binary indicators	n (or n = 1)	% (if binary)	​
SEROany	2,227	83.5	
Has polypharmacy	1,297	48.6	
High-risk PARS	671	25.2	

### 3.2 Operating threshold and primary model performance

For clinical screening, we evaluated fixed probability thresholds {0.3, 0.4, 0.5, 0.6} to convert model probabilities into binary decisions. As shown in Table 3, we selected 0.30 as the fixed operating threshold because it achieved the highest F1 for the high-risk class while preserving recall (precision 0.795, recall 0.808, F1 0.802, accuracy 0.908).

**TABLE 3:** Performance across probability thresholds for the High-risk PARS classifier (logistic regression). Metrics for precision, recall, and F1 refer to the positive class (High-risk PARS = 1).

Threshold	Accuracy	AUC	Precision (class 1)	Recall (class 1)	F1 (class 1)
**0.30**	**0.908**	**0.931**	**0.795**	**0.808**	**0.802**
0.40	0.902	0.931	0.822	0.733	0.775
0.50	0.900	0.931	0.886	0.650	0.750
0.60	0.896	0.931	0.912	0.608	0.730

Bold values indicate the selected threshold (0.30), which achieved the highest F1 score while preserving recall.

On the test set, the pre-specified threshold of 0.30 yielded the highest F1 and highest recall (see Table 3). In contrast, thresholds derived on the training set (F1-optimal = 0.410; Youden-optimal = 0.397) increased precision (0.830 and 0.822, respectively) but reduced recall (both at 0.733) and F1 (0.779 and 0.775, respectively). These results support 0.30 as a practical screening operating point.

**FIGURE 1:**
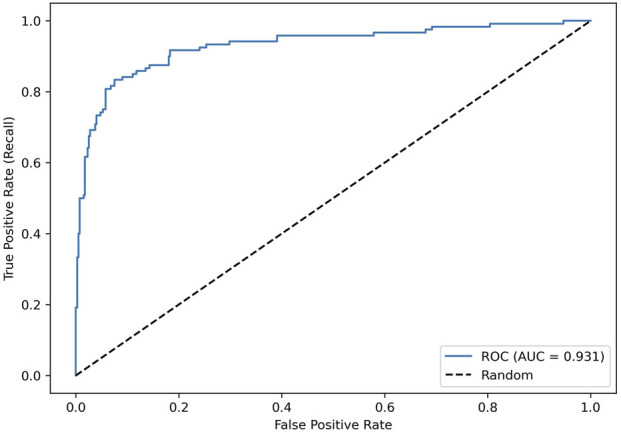
ROC curve of the primary logistic regression model trained on the patient-level 80–20 split stratified by whether a patient had ≥ 1 high-risk PARS visit; all visits from a patient assigned to a single split and evaluated on the held-out test set.

Figure 1 shows that the model showed strong threshold-independent discrimination (ROC-AUC = 0.931). AUC is threshold-independent, hence, values shown are identical across thresholds by definition.

### 3.3 PARS-based risk strata and binary alert (high-risk PARS)

**FIGURE 2:**
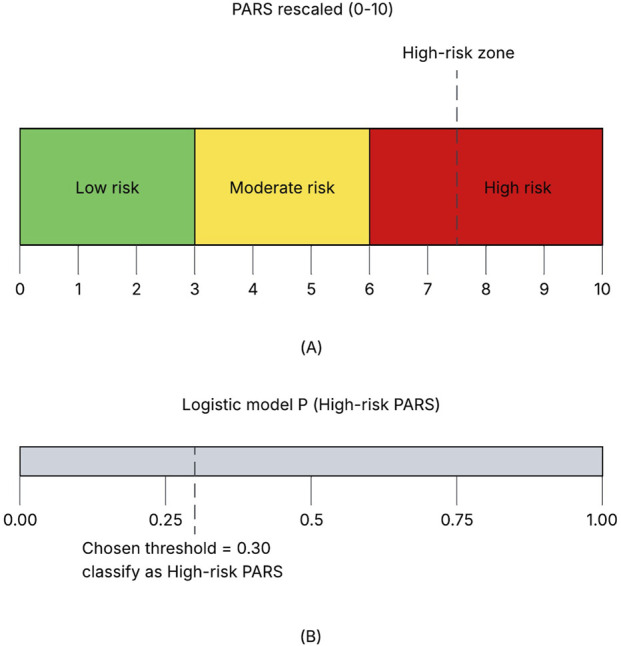
PARS rescaled strata and High-risk PARS decision rule for the primary drug-based logistic regression model (patient-level 80–20 split stratified by whether a patient had ≥ 1 High-risk PARS visit; all visits from a patient confined to a single split. **(A)** The continuous PARS rescaled score is mapped to qualitative bands on a 0–10 scale: low risk (0–3), moderate risk (3–6), and high risk (6–10). **(B)** Output of the drug-based logistic model shown as predicted probability of High-risk PARS (defined as PARS ≥τ 75); the vertical dashed line marks the pre-specified operating threshold of 0.30; visits with $\hat{p}\geq$ 0.3 are labeled as screen-positive (predicted High-risk PARS = 1, otherwise 0.).

Figure 2A shows that PARS rescaled provided a continuous 0–10 summary of pharmacological risk (median 3.19; IQR 2.53–4.12), which we organized into low, moderate, and high risk strata for clinical interpretation. To enable screening, the logistic model converted visit-level features into a probability of High-risk PARS and was operated at a fixed threshold of 0.30 (Figure 2B). At this operating threshold of 0.30, 25.2% of visits 31.0% of patients ( ≥ 1 flagged visit) were classified as High-risk in the whole cohort; in the held-out test set, 23.5% of visits were flagged as screen-positive, balancing precision and recall while preserving discrimination (AUC = 0.931).

### 3.4 Robustness across validation schemes

We present the comparative model performance in Table 4. Across validation schemes, discrimination remained stable, with an AUC ranging from 0.931 to 0.939, indicating robustness to the data splitting strategy. For the primary patient-level 80–20 split stratified by High-risk status, AUC was 0.931 with a patient-bootstrap 95% CI of 0.875–0.971; a visit-level bootstrap yielded a narrower CI of 0.900–0.959. Model calibration was acceptable: on the held-out test set, the Brier score was 0.0754 (Supplementary Figure S1), and the pooled out-of-fold Brier score for GroupKFold was 0.0812 (Supplementary Figure S2); together with the reliability diagrams, these results supported reasonable agreement between predicted probabilities and observed event rates.

**TABLE 4:** Comparative performance of three validation schemes for the High-risk PARS logistic classifier. Accuracy, precision, recall, and F1 are reported at the fixed clinical threshold of 0.30. Primary analysis uses patient-level 80–20 split stratified by High-risk status. Metrics for precision, recall, and F1 refer to the positive class (High-risk PARS = 1).

Model	Split strategy	AUC	Accuracy	Precision (1)	Recall (1)	F1 (1)
80–20, strat high-risk only	Train/test 80–20 at patient level; stratified by patient indicator of having ≥ 1 high-risk PARS visit	0.931	0.908	0.795	0.808	0.802
80–20, strat age + sex + High-risk	Train/test 80–20; combined stratification by age group ( < 65/ ≥ 65), sex (F/M), and high-risk PARS (yes/no)	0.938	0.878	0.754	0.781	0.767
5-Fold GroupKFold	5-Fold cross-validation grouped by patient (GroupKFold); all visits of a patient are confined to a single fold	0.939	0.883	0.749	0.818	0.781

At the prespecified operating point of 0.30, the primary 80–20 split stratified by High-risk status provided the most balanced trade-off, yielding the best F1 while maintaining high accuracy, with both precision and recall favorable for clinical screening. The 5-fold GroupKFold emphasized higher recall (greater case finding) with a modest reduction in precision. Additional stratification by age and sex did not improve overall F1. These results collectively support a threshold of 0.30 for screening high-risk PARS probability. This balance optimizes detection of high-risk cases while minimizing false positives, thereby reinforcing the reliability of the PARS-based alert rule.

Because polypharmacy (a drug-count indicator) and DDIscore (a severity-weighted interaction burden) may capture overlapping aspects of drug complexity, we assessed component overlap. Multicollinearity was limited (VIF 1.75 for polypharmacy and 1.96 for DDIscore; all component VIFs < 2), supporting stable estimation. Using quartile-based discordance criteria, high DDIscore ( ≥ Q75) occurred exclusively in polypharmacy visits (0/2,666 visits with < 5 drugs and DDIscore ≥ Q75), whereas polypharmacy with very low DDIscore ( ≤ Q25) was rare (8/2,666 visits, 0.30%). Overall, in this cohort, DDI burden is strongly influenced by drug count while remaining conceptually distinct as a severity-weighted interaction measure. Consistent with the VIF analysis, pairwise correlations among PARS components were generally moderate and did not indicate problematic collinearity (Supplementary Figure S3).

### 3.5 Drugs driving high-risk PARS

Table 5 summarizes the top 20 drugs associated with High-risk PARS (left; positive logistic coefficients, reported with odds ratios) and, for contrast, drugs with negative coefficients (right), which are associated with lower odds of High-risk PARS; for each drug, we also report the number of visits in which the drug appears (regardless of outcome). To reduce instability from rare exposures, we report only drugs with sufficient support (i.e., number of visits ≥ 20); under this criterion, the low-risk side includes 11 drugs that met the threshold. These coefficients quantify associations with the binary High-risk PARS outcome (not causation) and reflect average effects across co-medication and polypharmacy patterns in this cohort.

**TABLE 5:** Top drugs associated with high-risk PARS and low-risk PARS. Coefficients are from the logistic regression model; odds ratios are reported with 95% confidence intervals (patient-level bootstrap). Visits denote the number of cohort visits containing the drug. Few drugs met the inclusion criterion (Visits ≥20 ) with negative coefficients; therefore all eligible low-risk drugs are shown.

High-risk PARS drugs (coef > 0)			
Drug name	Coefficient	Odds ratio (95% CI)	Visits
Quetiapine	3.299	27.075 (16.856–43.183)	459
Haloperidol	2.850	17.290 (4.913–37.712)	67
Entacapone	2.661	14.304 (1.699–26.758)	37
Amiodarone	2.362	10.611 (1.028–20.445)	24
Trazodone	1.998	7.372 (3.937–11.176)	423
Venlafaxine	1.977	7.221 (3.229–11.170)	216
Risperidone	1.934	6.918 (3.008–11.406)	276
Tramadol	1.930	6.887 (3.133–10.829)	23
Clozapine	1.830	6.235 (1.493–16.009)	108
Trihexyphenidyl	1.731	5.645 (2.240–17.157)	130
Formoterol	1.711	5.536 (2.540–11.290)	73
Sertraline	1.705	5.502 (1.634–9.845)	92
Olanzapine	1.701	5.480 (2.435–9.581)	155
Bisoprolol	1.619	5.047 (2.655–10.827)	297
Rivaroxaban	1.616	5.033 (0.833–11.379)	21
Nebivolol	1.602	4.963 (2.810–8.783)	205
Escitalopram	1.594	4.925 (2.230–7.370)	272
Tiapride	1.594	4.924 (1.836–8.862)	74
Flupentixol	1.544	4.682 (0.714–10.908)	58
Acetaminophen	1.526	4.602 (2.206–7.056)	21

Low-risk PARS drugs (coef < 0)^a^			
Drug name	Coefficient	Odds ratio (95% CI)	Visits
Ivabradine	− 0.830	0.436 (0.250–1.324)	39
Piracetam	− 0.353	0.702 (0.247–2.002)	52
Amisulpride	− 0.347	0.707 (0.270–1.983)	52
Sitagliptin	− 0.263	0.769 (0.476–1.487)	34
Ramipril	− 0.255	0.775 (0.280–2.753)	46
Nitroglycerin	− 0.254	0.776 (0.369–1.602)	44
Rilmenidine	− 0.245	0.783 (0.326–2.553)	67
Doxepin	− 0.184	0.832 (0.573–1.000)	32
Alfacalcidol	− 0.109	0.896 (0.416–2.532)	53
Trimetazidine	− 0.057	0.944 (0.436–2.118)	127
Perindopril	− 0.019	0.981 (0.518–2.017)	316

^a^ Negative coefficients are reported for interpretability and transparency and do not imply protective effects or suggest medication changes; the actionable output is the model’s predicted High-risk probability.

The drug-only logistic model highlighted that central nervous system (CNS) drugs dominated the positive associations with High-risk PARS, led by antipsychotics (e.g., quetiapine, haloperidol, risperidone), antidepressants (e.g., trazodone, venlafaxine, sertraline), anti-Parkinson’s drugs (e.g., entacapone and trihexyphenidyl), and the opioid analgesic tramadol; alongside are cardiovascular drugs frequently co-prescribed in complex regimens (e.g., amiodarone, bisoprolol, nebivolol, and rivaroxaban). In contrast, the few drugs with negative coefficients and sufficient support were predominantly cardiovascular or cardiometabolic therapies (e.g., ivabradine, ACE inhibitors, antianginals, and antidiabetics). We report negative coefficients to characterize inverse associations and co-prescription patterns; these are not intended as actionable targets, and low-risk does not imply pharmacological protection.

### 3.6 Patient-level profiles of pharmacological risk

To complement drug- and visit-level analyses, we summarized patients ranked by (i) the number of High-risk PARS visits $\left(n_{\text{High}-\text{Risk}}\right)$ , which indicates the event burden, (ii) the maximum PARS rescaled observed per patient ( ${\text{PARSr}}^{\text{max}}$ ), representing the peak intensity, and (iii) the mean PARS rescaled across visits ( ${\text{PARSr}}^{\text{mean}}$ ), which reflects sustained exposure.

Table 6 lists the most affected patients ranked by the number of High-risk PARS visits, with de-identified ranks and age bands by decades. These patients typically had 11–12 visits and showed both high peak PARS rescaled and elevated mean PARS rescaled, indicating sustained pharmacological risk rather than isolated spikes. Most fall in the 60–79 years age range and include both sexes. Together, these profiles highlight candidates for targeted drug review and deprescribing, given their recurrent high-risk episodes and persistently high risk burden.

**TABLE 6:** Top 10 patients by burden of High-risk PARS $\left(n_{\text{High}-\text{risk}}\right)$ , with peak and mean PARS summaries. Patient identifiers are rank-only and de-identified.

Patient rank	Number of visits	PARSr^max^	PARSr^mean^	PARS^max^	PARS^mean^	$n_{\text{High}-\text{risk}}$	Sex	Age by decades
1	12	5.424	5.025	0.978	0.798	12	M	60–69
2	12	4.830	4.797	0.711	0.696	12	F	50–59
3	12	6.621	5.528	1.515	1.0241	12	M	60–69
4	12	7.183	6.069	1.767	1.267	12	F	60–69
5	12	6.569	6.044	1.492	1.256	12	F	40–49
6	12	5.889	5.369	1.186	0.953	12	M	70–79
7	11	5.230	5.063	0.890	0.815	11	F	70–79
8	11	5.700	4.884	1.101	0.735	11	M	70–79
9	12	6.210	5.624	1.330	1.067	11	F	70–79
10	11	6.173	5.625	1.314	1.068	11	F	70–79

Table 7 lists the top 10 patients ranked by the maximum PARS rescaled ( ${\text{PARSr}}^{\text{max}}$ ), which the pharmacological peak risk. This top shows pronounced peaks, with the leading patient reaching the maximum score of 10 and all the others exhibiting ${\text{PARSr}}^{\text{max}}$ between 6.512 and 7.591. Most of these patients also display elevated ${\text{PARSr}}^{\text{mean}}$ (5.931–8.354), indicating that peaks often occur on a high baseline rather than as isolated spikes. The event burden varies: some patients reach high peaks with relatively few visits (e.g., 3–7 visits), while others combine high peaks with frequent High-risk episodes (e.g., ranks with 11–12 visits and $n_{\text{High}-\text{Risk}}$ = 9–12). Age decades cluster mainly in the 50–79 years, in both sexes. Together, these profiles flag patients for targeted drug review: those with extreme peaks (safety concerns) and those with high peaks plus recurrent High-risk visits (sustained risk).

**TABLE 7:** Top 10 patients by maximum PARS rescaled ( ${\text{PARSr}}^{\text{max}}$ ), with total visits, mean PARS rescaled ( ${\text{PARSr}}^{\text{mean}}$ ), raw PARS maxima and means, number of High-risk PARS visits $\left(n_{\text{High}-\text{risk}}\right)$ , sex, and age range. Patients are de-identified by rank.

Patient rank	Number of visits	PARSr^max^	PARSr^mean^	PARS^max^	PARS^mean^	$n_{\text{High}-\text{risk}}$	Sex	Age by decades
1	11	10	8.354	3.033	2.294	11	F	50–59
2	6	7.591	6.291	1.951	1.367	6	M	70–79
3	7	7.561	5.928	1.937	1.204	6	F	50–59
4	12	7.183	6.069	1.767	1.267	12	F	60–69
5	11	6.771	5.143	1.582	0.851	9	M	80–89
6	12	6.621	5.528	1.515	1.024	12	M	60–69
7	4	6.616	5.564	1.513	1.040	4	M	60–69
8	3	6.571	5.851	1.493	1.169	3	F	60–69
9	12	6.569	6.044	1.492	1.256	12	F	40–49
10	4	6.512	5.931	1.466	1.205	4	F	70–79

Table 8 lists the first 10 patients ranked by ${\text{PARSr}}^{\text{mean}}$ , which illustrates the sustained pharmacological risk. Patients exhibit consistently elevated risk across visits: the leading patient averages ${\text{PARSr}}^{\text{mean}}$ of 8.354, with 11 of 11 visits categorized as high-risk. The other patients maintain ${\text{PARSr}}^{\text{mean}}$ between 5.851 and 6.291 with frequent High-risk episodes (typically 6–12 visits with many classified as high-risk). Peaks scores are high (between 6.571 and 10), showing that the high means are driven by the pronounced maximum episodes. Age decades cluster in the 50–79 years across both sexes. These profiles highlight patients with chronic high PARS exposure, who may benefit most from longitudinal drug review and targeted deprescribing efforts.

**TABLE 8:** Top 10 patients by mean PARS rescaled ( ${\text{PARSr}}^{\text{mean}}$ ), with total visits, maxima PARS rescaled ( ${\text{PARSr}}^{\text{max}}$ ), raw PARS maxima and means, number of High-risk PARS visits $\left(n_{\text{High}-\text{risk}}\right)$ , sex, and age decades. Patients are de-identified by rank.

Patient rank	Number of visits	PARSr^max^	PARSr^mean^	PARS^max^	PARS^mean^	$n_{\text{High}-\text{risk}}$	Sex	Age by decades
1	11	10	8.354	3.033	2.294	11	F	50–59
2	6	7.591	6.291	1.951	1.367	6	M	70–79
3	1	6.252	6.252	1.350	1.350	1	F	70–79
4	12	7.183	6.069	1.767	1.267	12	F	60–69
5	1	6.047	6.047	1.257	1.257	1	M	40–49
6	12	6.569	6.044	1.492	1.256	12	F	40–49
7	1	5.932	5.932	1.206	1.206	1	F	80–89
8	4	6.512	5.931	1.466	1.205	4	F	70–79
9	7	7.561	5.928	1.937	1.204	6	F	50–59
10	3	6.571	5.851	1.493	1.169	3	F	60–69

### 3.7 Top high-risk visits

To characterize high-severity episodes in terms of pharmacological risk, Table 9 lists the top 10 high-risk visits ranked by PARS rescaled. For each visit, we report de-identified demographics (sex, age decade), the whole drug regimen as prescribed at that encounter, and the resulting PARS rescaled. Because several patients shared identical regimens (and thus identical scores) during multiple visits, we retained one representative record per unique regimen when appropriate to avoid redundancy and enhance the table’s readability.

**TABLE 9:** Top 10 high-risk visits ranked by PARS rescaled (PARSr), with de-identified demographics and whole drug regimens.

Visit rank	Sex	Age decade	Drug regimen	PARSr
1	F	50–59	Flupentixol, clozapine, trihexyphenidyl, haloperidol, zolpidem, diazepam, alprazolam, fenofibrate, nebivolol, tibolone, atorvastatin, oxybutynin, alfacalcidol, beclomethasone dipropionate, formoterol, levocetirizine	10
2	F	50–59	Clozapine, flupentixol, trihexyphenidyl, haloperidol, diazepam, alprazolam, fenofibrate, nebivolol, tibolone, atorvastatin, oxybutynin, alfacalcidol, beclomethasone dipropionate, formoterol, levocetirizine	9.456
3	F	50–59	Clozapine, flupentixol, carbamazepine, trihexyphenidyl, haloperidol, zolpidem, alprazolam, diazepam, fenofibrate, nebivolol, tibolone, atorvastatin, oxybutynin, alfacalcidol	9.110
4	F	50–59	Flupentixol, clozapine trihexyphenidyl, haloperidol, alprazolam, diazepam, sitagliptin, metformin, fenofibrate, nebivolol, tibolone, atorvastatin, oxybutynin, alfacalcidol, beclomethasone dipropionate, formoterol, levocetirizine	8.127
5	M	70–79	Valproic acid, zopiclone, quetiapine, buspirone, alprazolam, lorazepam, levodopa, benserazide, trihexyphenidyl, betahistine, saxagliptin, dapagliflozin, metformin	7.591
6	F	50–59	Clozapine, valproic acid, trihexyphenidyl, nitrazepam, lorazepam, trimetazidine, desloratadine, trospium, budesonide, formoterol, omeprazole, bisoprolol	7.561
7	F	50–59	Flupentixol, clozapine, trihexyphenidyl, haloperidol, fenofibrate, nebivolol, tibolone, atorvastatin, oxybutynin, alfacalcidol, metformin	7.404
8	F	50–59	Flupentixol, clozapine trihexyphenidyl, haloperidol, alprazolam, diazepam, fenofibrate, nebivolol, tibolone, atorvastatin, oxybutynin, alfacalcidol, beclomethasone dipropionate, formoterol, levocetirizine	7.361
9	M	70–79	Valproic acid, buspirone, quetiapine, lorazepam, alprazolam, saxagliptin, dapagliflozin, metformin, nicergoline, levodopa, benserazide, trihexyphenidyl, betahistine	7.343
10	F	60–69	Quetiapine, haloperidol, trihexyphenidyl, diazepam, lorazepam, furosemide, fenofibrate, nebivolol, omeprazole, amoxicillin	7.183

The highest ranked visits are characterized by intense polypharmacy and CNS-heavy combinations, typically antipsychotics (clozapine, haloperidol, flupentixol, quetiapine) paired with benzodiazepines and Z-drugs (alprazolam, diazepam, lorazepam, zolpidem, zopiclone) and adjunctive drugs to manage extrapyramidal symptoms (e.g., trihexyphenidyl) and mood stabilizers (e.g., valproic acid). Many regimens also include cardiometabolic or respiratory therapies (e.g., nebivolol, atorvastatin, fenofibrate, metformin, saxagliptin, formoterol, beclomethasone dipropionate), reflecting severe multimorbidity. Most visits occur in mid-to-late adulthood (50–79 years). These examples translate the overall findings into case-level patterns that are immediately actionable for drug review, highlighting combinations where deprescribing or mitigating DDIs could provide the most significant safety improvements.

## 4 Discussion

### 4.1 Cohort description and component score distribution

As presented in Table 1, our cohort reflects a busy ambulatory psychiatry service, with 2,666 visits from 680 adults, and with balanced representation throughout middle and older age decades, along with a female predominance, which is typical for outpatient mental health settings. We highlight two clinically relevant prevalence figures: 31% of patients who experienced at least one High-risk visit during follow-up (Table 1) and 25.2% of visits with High-risk PARS at the pre-specified 0.30 threshold (see Table 2). These figures suggest that pharmacological risks are often present in the prescribing stage, highlighting the need for a screening tool integrated into routine workflows.

The pharmacological risk component scores exhibited an uneven distribution—many were low, while only a few were very high—consistent with clinical practice, as shown in Table 2. The DDIscore is right-skewed, with many low-moderate values and a long tail reflecting complex regimens. ACB score visit showed a similar pattern, primarily occurring at low to intermediate levels; however, we observed a significant upper tail: 41% of visits recorded an ACB ≥ 3, a threshold often associated with cognitive adverse consequences in elderly patients. AZCERTmax was primarily concentrated in categories 1 and 2, while category 3, which indicates a known risk of TdP, was less frequent but still clinically significant. The high prevalence of SEROany score, capturing whether any serotonergic drug was present, indicates that serotonergic exposure is common in our cohort; this result aligns with the routine prescribing of serotonergic psychotropic drugs (e.g., SSRIs, SNRIs, and related agents). Polypharmacy occurred in about half of the visits, which is consistent with outpatient psychiatry.

### 4.2 Model performance and robustness

The results presented in Table 4 highlight that the logistic model consistently showed stable performance in identifying high-risk PARS visits, demonstrating that the performance remained robust regardless of how the data were partitioned; similar AUC values obtained in the three split strategies suggest that model discrimination is not driven by a favorable single split and that the learned signal persists under repeated resampling. At the same time, with the operating threshold set at 0.30 (chosen to prioritize case-finding in a screening context), the threshold-dependent metrics remained clinically satisfactory and consistent across validation strategies (see Tables 3, 4).

Two methodological aspects strengthen confidence in these findings. First, all evaluations used patient-level partitioning, which prevents information leakage between training and test sets in a dataset with repeated visits per individual. Second, the close agreement in AUC and operating-point metrics across the primary holdout split, the more restrictive age and sex stratified split, and patient-grouped 5-fold cross-validation indicates that the results are not driven by a particular subset of patients or by a favorable partition, and the model is not too sensitive to cohort composition. Nonetheless, some spread in threshold-dependent measures is expected, especially for infrequently prescribed drugs; this highlights the importance of reporting feature support (e.g., number of visits) and emphasizes the need to use a prespecified decision threshold and grouped validation when converting PARS probabilities into clinically actionable alerts.

### 4.3 Drugs driving high-risk PARS: which drugs matter

Table 5 reveals that the logistic model provides a consistent clinical pattern regarding drugs most associated with High-risk PARS visits, in particular, drugs acting on the central nervous system dominated the positive associations. The strongest signals were for antipsychotics (e.g., quetiapine and haloperidol), followed by antidepressants (e.g., trazodone, venlafaxine, sertraline) and anti-Parkinson drugs (e.g., entacapone and trihexyphenidyl). The clinical coherence of this pattern is explained by their frequent concomitant occurrence in complex regimens, often being linked to a higher anticholinergic load, QT susceptibility, serotonergic exposure, or increased DDI profiles, all of which contribute to the composite PARS signal Gatti et al. (2021); Hjorth (2021); Parabiaghi et al. (2024).

Beyond psychotropics, several non-psychiatric agents commonly used in multimorbid patients showed positive associations, including cardiovascular drugs (e.g., amiodarone, bisoprolol, and nebivolol) and analgesics (e.g., tramadol and acetaminophen) (see Table 5). The presence of these drugs may indicate clinical complexity, including cardiometabolic comorbidities and polypharmacy, rather than the isolated pharmacodynamic effects of any single agent Gebresillassie and Kassaw (2023); Gundugurti et al. (2022); Radu et al. (2020). Therefore, one should interpret the coefficients as conditional associations based on the observed prescribing patterns, not as causal risk contributions attributable to individual drugs. Reporting the support features, such as the number of visits in which each drug appeared, helps contextualize these estimates and reduces overinterpretation of rare exposures.

The small set of drugs with negative coefficients predominantly includes cardiovascular or cardiometabolic drugs (e.g., ivabradine, sitagliptin, ramipril, nitroglycerin, rilmenidine, perindopril), which likely indicates that specific prescribing profiles—often involving fewer psychotropics or lower anticholinergic, QT, and serotonergic load—were less frequently labeled as High-risk. Notably, low-risk in this context does not indicate a protective pharmacological effect, but rather serves as a marker of regimen patterns that tend to carry a lower composite PARS signal in this dataset.

These results analyzed from a clinical decision support perspective suggest a prioritization strategy: when High-risk PARS is flagged, the most efficient approach to drug review may be to start with CNS-acting agents that frequently appear on the high-association list (Table 5); the next step should be to identify additive risk mechanisms (e.g., anticholinergic overlap, QT risk combinations, serotonergic clustering, and polypharmacy). This strategy aligns the model’s interpretable outputs with actionable steps for deprescribing and monitoring, while ensuring caution about confounding caused by indication and co-prescription structure in observational data Jerjes et al. (2024); Garfinkel et al. (2015); Tchijevitch et al. (2025).

### 4.4 Patient-level risk profiles

Patient-level rankings provided in Tables 6–8 offer a complementary perspective to visit-level performance by illustrating how pharmacological risk concentrates within individuals over time. Within these summaries, a small subset of patients encountered repeated High-risk PARS, typically combining both a high frequency of visits and a consistently elevated PARS rescaled. This pattern suggests that High-risk PARS is not just an occasional phenomenon; for some patients, it represents an ongoing prescribing profile; these patients may benefit from structured drug reconciliation, pharmacist-led review, and long-term deprescribing plans rather than single-visit adjustments.

Since we calculated PARS rescaled for each visit, repeated measures per patient visit may provide valuable clinical insights. The distinction between maximal and average rescaled PARS further clarifies clinically relevant phenotypes. Patients with episodic peaks (high ${\text{PARS}}^{\text{max}}$ ) indicate visits in which risk components aligned simultaneously (e.g., high DDI burden, high anticholinergic load, QT-liability, serotonergic exposure, and polypharmacy) (Table 7). In contrast, patients with high ${\text{PARS}}^{\text{mean}}$ show sustained peaks across visits, consistent with chronic exposure to complex drug regimens and cumulative risk mechanisms (see Table 8). Notably, top-ranked ${\text{PARS}}^{\text{mean}}$ patients tend to also have high ${\text{PARS}}^{\text{max}}$ , suggesting that sustained high-risk profiles often occur on a background of episodic extremes rather than stable moderate burden alone. This structure underscores the need for longitudinal stewardship, such as drug reconciliation and deprescribing, instead of relying solely on one-off interventions.

Demographic summaries (sex and age by decades) indicate that high-risk profiles appear across multiple age decades and in both sexes, confirming that the PARS framework does not capture a single demographic risk factor but the complexity of the regimen (Tables 6–8) Alsfouk et al. (2025). From an implementation standpoint, these patient-level patterns are directly actionable. Patients with high $n_{\text{High}-\text{risk}}$ represent candidates for proactive, longitudinal drug optimization. Patients with high ${\text{PARS}}^{\text{max}}$ may require a rapid review of the specific high-risk visit regimen to identify modifiable drivers (e.g., anticholinergic stacking, QT-risk combinations, or serotonergic clustering). In this way, patient-level profiling complements visit-level screening by helping physicians to prioritize follow-up frequency and freeing up pharmacists’ time for those patients most likely to benefit from repeated intervention.

### 4.5 High-risk visits: actionable patterns

Table 9 illustrates that the highest PARSr values result from multiple culprit drugs and the overlap of several clinically plausible risk domains in the same visit: dense polypharmacy, high DDI opportunity, additive anticholinergic cognitive burden, QT-susceptible drug combinations, and frequent serotonergic exposure. This pattern is in line with the clinical reality, where patients with severe mental illness often have substantial somatic comorbidities (particularly cardiometabolic and respiratory diseases) and receive complex drug treatment, leading to fragmentation of supervision (De Hert et al., 2011; Dornquast et al., 2017). It is noteworthy that several top visits present very similar drug regimens, thus suggesting that high pharmacological risk is often recurrent and sustained; therefore, they may benefit from longitudinal drug review instead of only episodic, incidental alert resolution. Because electronic drug interaction checkers may classify the same drug pair with a discrepant severity level, DDI surveillance should be a structured decision support that still requires further clinical analysis and documentation. This limitation becomes more relevant as polypharmacy increases and inconsistency between databases is more likely; this reinforces the need for systematic review workflows instead of relying on a single checker Suciu et al. (2024); Colibăşanu et al. (2025).

Across the top-10 visits presented in Table 9, the psychotropic core is characterized by antipsychotic polypharmacy and sedatives; it frequently includes combinations of antipsychotics (e.g., clozapine, haloperidol, and flupentixol), adjunct anticholinergics (e.g., trihexyphenidyl), and sedative-hypnotics (e.g., zolpidem, diazepam, alprazolam, and lorazepam). This combination of drugs is clinically relevant: anticholinergics help to manage extrapyramidal symptoms, while benzodiazepines and hypnotics address agitation, anxiety, and sleep disorders. However, when used together, these drugs can increase the risk of cognitive burden, falls, sedation, and functional decline—precisely the type of cumulative risk that PARS is designed to identify. Clozapine-containing regimens are particularly relevant for clinical discussion. Clozapine is an antipsychotic with many advantages for the psychiatric patient. However, it is considered a reserve drug due to its difficult-to-tolerate side effects (sedation, weight gain, sialorrhea, agranulocytosis, tachycardia, and other cardiovascular disorders) Martini et al. (2021). Clozapine also has a high potential for drug interactions, as it can interact with other drugs metabolized by CYP3A4, and even with cigarette smoke, which can induce CYP1A2 (in smokers, the concentration of clozapine can be reduced) De Berardis et al. (2018); Preskorn (2018).

The anticholinergic dimension visible in these top visits is highlighted by drugs such as clozapine (with an ACB score of 3, which means a significant risk of cognitive and metabolic impairment), haloperidol (ACB score of 2 and with an increased risk of extrapyramidal syndrome), diazepam and alprazolam (ACB score of 1 and risk of sedation and development of dependence), trihexyphenidyl and oxybutynin (both with an ACB score of 3) King and Rabino (2024). This way, the concomitant use of these drugs may lead to high ACB accumulation and associated cognitive and sedation burden. Therefore, reducing the anticholinergic score may benefit the patient with schizophrenia, reducing delirium and cognitive impairment. The deprescribing of drugs with anticholinergic activity is essential for the overall functionality of the body, avoiding constipation, urinary retention, cognitive impairment, and sedation, being clinically meaningful for patients with severe mental illness Pjevac and Korošec Hudnik (2023); Lupu et al. (2021).

AZCERT lists haloperidol as having a known risk of QT prolongation, while clozapine and flupentixol are listed as having a possible risk; this highlights the concern of antipsychotic polypharmacy and the cumulative risk of severe ventricular arrhythmias Lambiase et al. (2019). There are also clinical situations where the QT interval may be altered by factors such as hypokalemia, hypomagnesemia, congenital heart disease, or heart failure Sankaranarayanan et al. (2024); Barbui et al. (2016).

Several high-ranked visits presented in Table 9 reveal combined psychotropics with cardiovascular and cardiometabolic drugs (e.g., beta-blockers, hipolipemiants, antidiabetics). These complex clinical contexts are typical in severe mental illness, given the elevated baseline risk of metabolic syndrome driven by both illness-related factors and drug effects De Hert et al. (2011); Kumar and Sidana (2017); Abavana and Sadiq (2025). The combination of statins and fibrates for dyslipidemias, may increase the risk of liver and kidney toxicity, as well as the risk of rhabdomyolysis Padilla-Padilla et al. (2025); Sobukawa et al. (2024). Furthermore, combining statins with saxagliptin may further increase the risk of rhabdomyolysis Labat et al. (2017); Montastruc (2025). Psychiatric patients are at higher risk of developing rhabdomyolysis Lu et al. (2025).

Some of the regimens in Table 9 contain the mood stabilizer, valproic acid, a fatty acid drug used off-label in bipolar affective disorder, including acute bipolar depression. It has known hepatotoxic effects, as well as its metabolites are associated with liver toxicity. Additionally, valproic acid can exacerbate liver damage in patients with metabolic syndrome Ezhilarasan and Mani (2022); Fleming and Chetty (2006). Co-administered drugs may influence the therapeutic range of valproic acid Bentué-Ferrer et al. (2010)—for example, quetiapine may decrease the plasma concentration of valproic acid Winter et al. (2007).

### 4.6 Practical implications for using PARS in clinical practice

The clinical interpretation of high-risk PARS visits is that they involve complex drug treatments, which are often necessary and plausible, but require prioritized review due to the intersection of multiple risk dimensions. A practical implication of the workflow is to prioritize interventions in the same order as PARS is structured: (1) reconcile the complete medication list, including acute drugs, (2) identify potentially avoidable sedative duplication and anticholinergic stacking, (3) evaluate QT risk and monitoring needs in context, and (4) assess high-risk DDIs and options for simplification. This actionable pattern could support patient-safe discussions, but the added value of PARS beyond single-domain alerts needs to be confirmed in prospective studies.

### 4.7 Limitations and future research

We should acknowledge several limitations of our study. First, our study lacked independent clinical outcomes (e.g., adverse drug events, ECG abnormalities, falls, or delirium) and was based on a single-center outpatient cohort; future studies should assess clinical validity and real-world utility using prospectively collected outcomes in external cohorts.

Second, the DDI component depends on the completeness and versioning of the underlying interaction knowledge base (here, DrugBank), which evolves over time. Recent work highlights the need for benchmarking and standardizing DDI severity to improve the practical utility of drug interaction resources Udrescu et al. (2023). Future work should quantify the DDI component’s contribution (e.g., via ablation analyses) and assess whether consensus DDI scoring across multiple resources improves PARS robustness and transportability. From an implementation perspective, high-risk signals may recur across visits and should support structured, longitudinal drug review, particularly given inter-database variability in DDI severity classification Suciu et al. (2024); Colibăşanu et al. (2025).

Third, PARS does not include a dedicated sedative burden component. Although benzodiazepines and Z-drugs contribute to anticholinergic cognitive burden as possible-risk agents, reflecting sedative effects, tolerance, and dependence, this does not fully capture cumulative sedative load. Incorporating a specific sedative burden feature represents an important direction for future refinement of PARS, especially in psychiatric populations.

Smoking is another relevant factor that was not systematically captured in our dataset. Smoking is highly prevalent in psychiatric populations and is associated with substantial respiratory morbidity (including COPD), which may be relevant when interpreting visits with respiratory therapies in this cohort Lores et al. (2018); Wu et al. (2025); Zheng et al. (2025). Smoking may also influence psychiatric drugs through pharmacokinetic mechanisms (e.g., induction of drug-metabolizing enzymes by constituents of tobacco smoke), while nicotine can pharmacodynamically alter treatment response and tolerability independent of drug concentrations Pan et al. (1999); Desai et al. (2001); Zevin and Benowitz (1999). We therefore consider smoking status and its intensity a critical variable for future work and external validation, both as a potential confounder and as a factor that may help differentiate regimen-based potential risk signals from clinically consequential medication-related problems.

Finally, given that PARS aggregates drug-risk domains frequently encountered in older adults, it may also be helpful as a screening tool in general geriatric populations, which warrants evaluation in broader cohorts. In addition, future studies could explore whether pharmacogenetic profiling in selected high-risk patients improves discrimination between potential medication-related risk signals and clinically consequential adverse outcomes, and quantify its incremental value when added to PARS Bousman et al. (2021); Sharew et al. (2024).

## 5 Conclusion

PsychoPharm Aggregated Risk Score (PARS) is a prioritization and decision-support metric that identifies drug regimens most likely to benefit from structured drug review. PARS is intended as a cohort-relative screening signal, not a calibrated estimate of absolute risk. By integrating five key pharmacological risk components—DDI burden, anticholinergic load, serotonergic exposure, polypharmacy, and QT-prolongation liability—into a single interpretable output at the visit level, PARS can prioritize complex regimens for structured review and drug reconciliation, particularly at the intersection of psychiatric and somatic prescribing. Thus, PARS can help clinicians and pharmacists to rapidly identify actionable opportunities, such as avoiding duplicate sedatives, reducing unnecessary anticholinergic stacking, and reassessing QT-relevant combinations arising from the intersection of psychiatric and somatic prescribing. In clinical practice, PARS may support drug reconciliation, highlight newly introduced interaction opportunities, and strengthen communication across prescribers and care settings. Overall, our approach is consistent with pharmacovigilance evidence indicating that clinically relevant DDIs often occur in psychiatric patients who are under antipsychotic polypharmacy and have concurrent somatic drugs. In this context, PARS may offer a pragmatic, integrated screening tool that complements single-domain assessments and helps prioritize drug review and identify candidates for structured deprescribing in high-risk psychiatric regimens.

## Data Availability

The data analyzed in this study is subject to the following licenses/restrictions: The datasets for this article are not publicly available due to concerns regarding patient anonymity. Requests to access the datasets should be directed to the corresponding authors. Requests to access these datasets should be directed to sabina.vasii@umft.ro; udrescu.lucretia@umft.ro.
