# Supplementary material for: PsychoPharm aggregated risk score (PARS): a multidimensional tool to flag high-risk pharmacotherapy in psychiatric patients
Source: Front Pharmacol. 2026 Apr 1;17:1782968. doi: 10.3389/fphar.2026.1782968 (PMC13079708; doi:10.3389/fphar.2026.1782968)
Supplement: Supplementary file 1 [file DataSheet1.pdf]

Supplementary Material

SUPPLEMENTARY METHODS

We provide additional diagnostics to support model calibration and analyze component overlap.

Calibration was evaluated using reliability diagrams with quantile-based binning (10 bins), where we plotted the observed event rates against mean predicted probabilities within each bin; the diagonal line on the graph indicates perfect calibration. For GroupKFold method, calibration curves were computed from pooled out-of-fold predictions to avoid optimistic bias.

Additionally, we summarized component overlap among PARS domains using a Spearman correlation matrix computed at the visit level to accommodate ordinal and binary components.

Figures

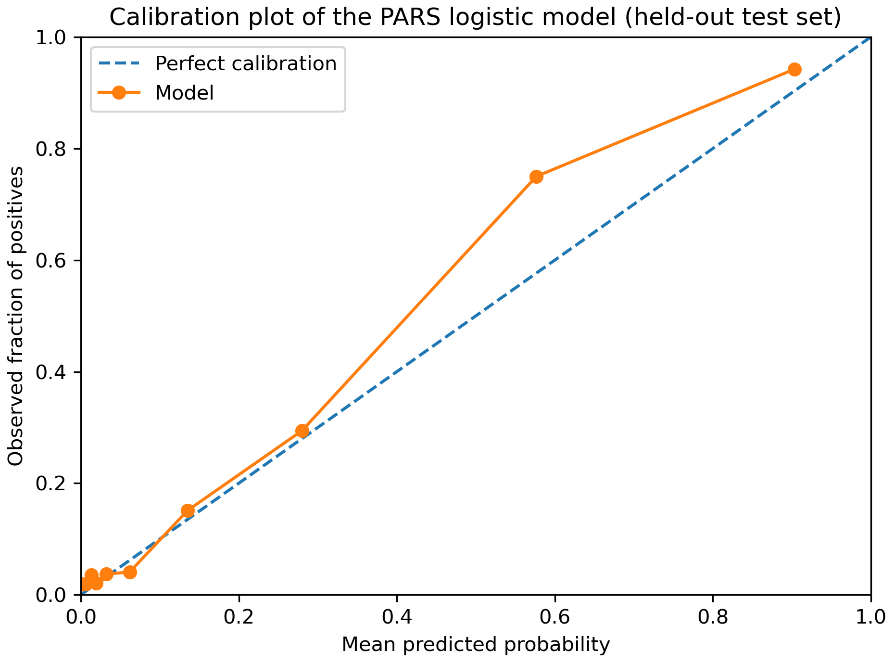

Figure S1. Calibration plot (reliability diagram) of the primary PARS logistic model on the held-out test set. Points represent observed event rates within quantile-based probability bins (10 bins) plotted against mean predicted probabilities; the dashed diagonal indicates perfect calibration.

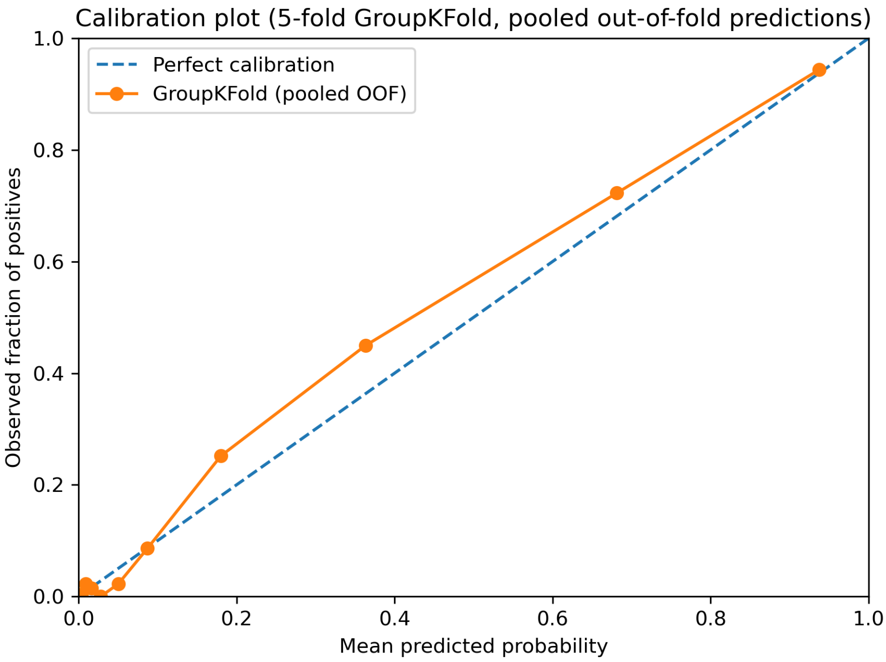

Figure S2. Calibration plot (reliability diagram) for the 5-fold GroupKFold scheme, computed from pooled out-of-fold predictions to avoid optimistic bias.

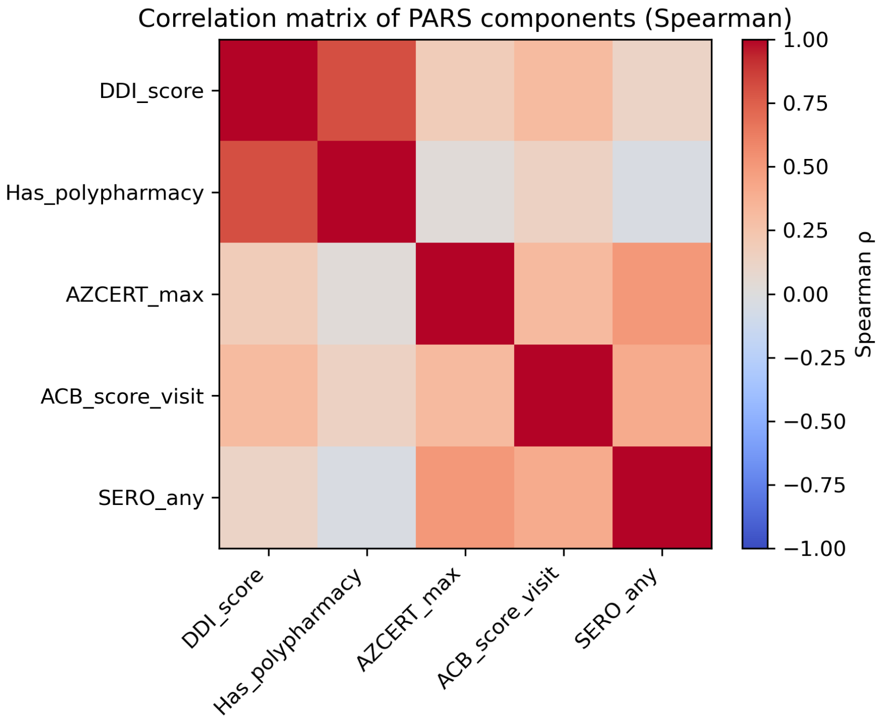

Figure S3. Spearman correlation matrix of PARS components computed at the visit level, including drug-drug interaction burden (DDI score), polypharmacy status (Has polypharmacy), maximum AZCERT risk category (AZCERT max), anticholinergic burden per visit (ACB score visit), and serotonergic exposure (SERO any). Spearman's rank correlation was used to account for ordinal and binary components, supporting the low multicollinearity observed in VIF diagnostics.
